# Evaluating synergistic effects among multiple factors in disease causation: a new approach using a generalized synergy index

**DOI:** 10.1007/s10654-026-01405-2

**Published:** 2026-05-29

**Authors:** Giuseppe La Torre, Pierpaolo D’Urso

**Affiliations:** 1 Department of Public health and Infectious Diseases, Sapienza University of Rome, Rome, Italy; 2 Department of Social Sciences and Economics, Sapienza University of Rome, Rome, Italy

**Keywords:** Synergy index, Disease causation, Additive interaction

## Abstract

The methodological objective of the present study is to extend Rothman’s additive interaction framework from two factors to three or more factors considered simultaneously, while preserving its original causal interpretation and operational simplicity. we propose a generalized Synergy Index (*S*_*p*_) that retains the additive logic of Rothman’s original formulation while extending it to an arbitrary number of dichotomous factors. For the analysis we used a database of a previous work in which the synergistic factor was calculated for each couples of two factors (alcohol, tobacco smoke and other risk factors) for age-related macular degeneration. The proposed three-factor *S*_*p*_ is sensitive to the underlying structure of the risk factors considered. When a strong susceptibility component such as family history is included, the combined exposure exhibits clear super-additive behavior (*S*_*p*_ = 1.783). Conversely, when hypercholesterolemia replaces family history, the joint effect remains substantial but does not exceed additivity (*S*_*p*_ = 0.756). The generalized multi-factor Synergy Index represents a valuable conceptual and analytical tool for investigating higher-order interactions. It is particularly well suited for the study of complex diseases, where multiple exposures co-occur and interact. It enables the identification of exposure constellations characterized by true superadditivity, with potential implications for etiological research, risk stratification, and targeted prevention strategies.

## Introduction

Synergism derives from the Greek term *synergos*, meaning “working together.” In scientific contexts, it describes the interaction between two or more agents in which the combined effect exceeds the sum of their individual effects. This phenomenon represents a form of supra-additivity, often summarized as a “more-than-additive” or “1 + 1 > 2” outcome [1].

Rothman conceptualizes synergy as the relationship between causal factors whose combined effect exceeds the sum of their individual effects [2–4]. Within this framework, the factors participating in a synergistic interaction are considered component causes of the same sufficient cause, defined as a complete set of conditions that inevitably produces a given outcome. Synergism therefore reflects a supra-additive interaction in which the joint influence of multiple factors substantially surpasses the simple aggregation of their independent contributions.

From a quantitative perspective, synergism in epidemiological or statistical modeling is typically inferred when the synergy index (S) exceeds 1, indicating a superadditive effect. Rothman’s “causal pie” model further illustrates this concept by depicting each causal factor as a necessary “slice” contributing to the completion of a sufficient cause [5]. Although the model can be extended to incorporate more than two causal components, it does not inherently provide a direct quantitative metric for measuring the magnitude of synergy.

According to the Rothman’s original method, the possible interaction between two variables can be calculated as follows: S = [RR_11_ − 1]/[(RR_01_ + RR_10_) − 2], where RR_11_ is equal to RR of the joint effect of two risk factors and RR_10_ and RR_01_ are equal to RR of each risk factor in the absence of the other. A value of S equal to 1 can be interpreted as indicative of additivity, whereas a value greater than 1 is indicative of superadditivity and synergism [2]. However, the possibility of the synergism between three or more factors has not been described.

More recently, Cortina-Borja and coll [6]. defined the synergy factor (*SF) a*s:

$$SF\,=\,O{R_{12}}/(O{R_1} \times O{R_2})$$

In this case SF is the ratio of the observed *OR* for both factors combined, to the predicted *OR* assuming independent effects of each factor. Among the limitation of this approach, Cortina-Borja and coll. reported that if a third interacting factor is present, and a sufficiently large dataset is available, *SF* analysis must be performed twice, after stratification by the third factor [6].

Contemporary epidemiological research increasingly confronts etiological scenarios characterized by the simultaneous presence of multiple behavioral, genetic, environmental, and metabolic determinants. In such contexts, limiting interaction assessment to pairwise comparisons may obscure higher-order causal mechanisms that only emerge when several factors are jointly present.

The methodological objective of the present study is therefore to extend Rothman’s additive interaction framework from two factors to three or more factors considered simultaneously, while preserving its original causal interpretation and operational simplicity.

## Methods

### Methodological need for generalization beyond two factors

While Rothman’s index is theoretically extensible, its explicit formulation for more than two factors has not been previously developed. As a result, epidemiological studies assessing multiple interacting exposures have typically relied on:

- Multiplicative interaction terms in regression models, or
- Stratified pairwise analyses repeated across levels of additional factors.

These approaches, although informative, do not provide a unified, additive-scale measure of higher-order synergism and become increasingly difficult to interpret as the number of factors grows.

The present methodological development addresses this gap by proposing a generalized Synergy Index that retains the additive logic of Rothman’s original formulation while extending it to an arbitrary number of dichotomous factors.

#### Exposure space and combinatorial structure

Let $\:p\:$denote the number of dichotomous exposure factors under investigation. Each factor can assume two possible states (0 = absent, 1 = present), generating a total of $\:{2}^{p}$distinct exposure configurations.

These configurations can be partitioned into three conceptual groups:

1. The null configuration $\:\left(\mathrm{0,0},\dots\:,0\right)$, representing complete absence of exposure;
2. The full configuration $\:\left(\mathrm{1,1},\dots\:,1\right)$, representing simultaneous exposure to all factors;
3. The remaining partial configurations, representing all possible cases in which at least one but not all factors are present.

**Fig. 1:**
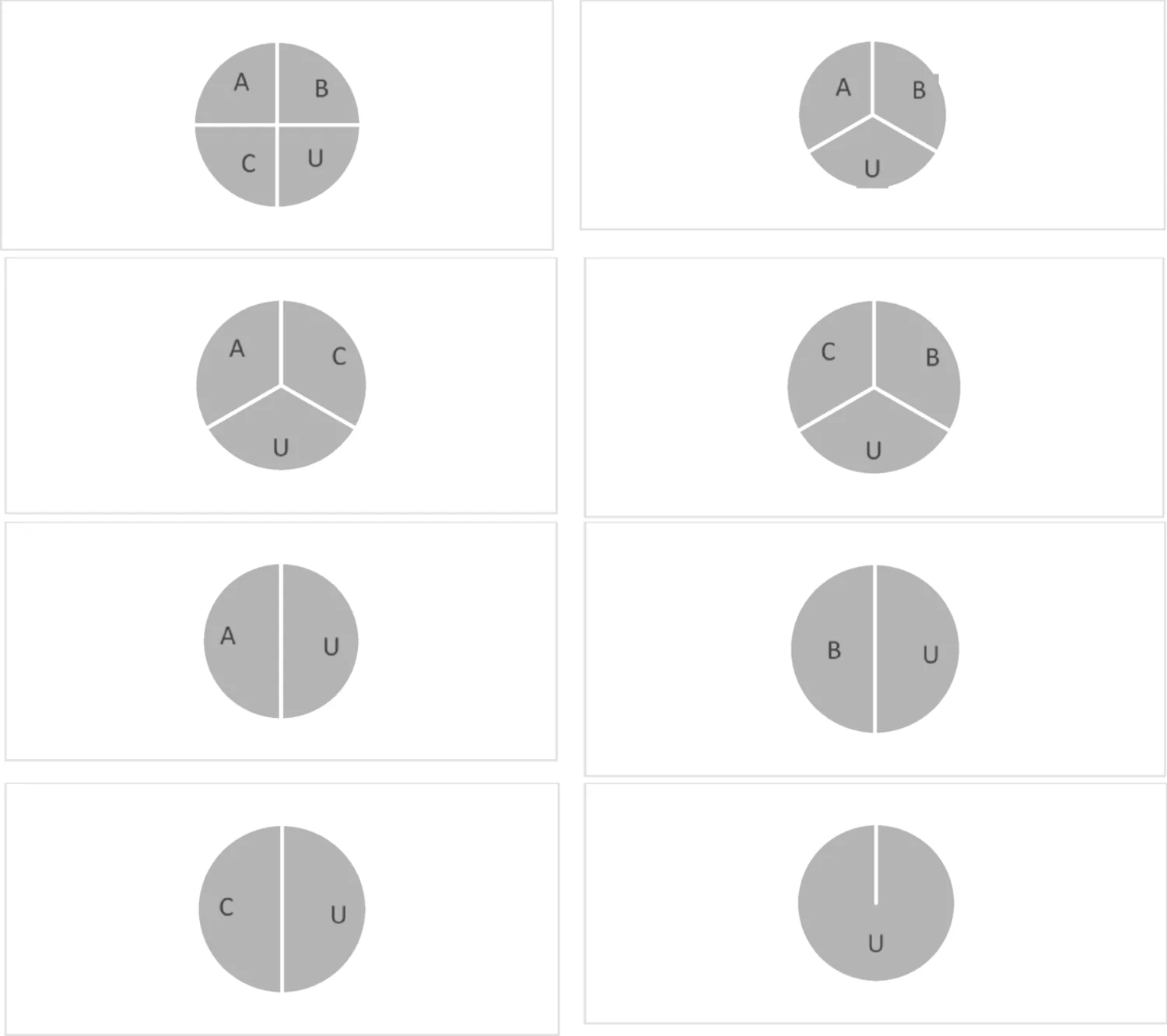
Eight classes of causal mechanisms in which the casual components A, B and C are involved

In Fig. 1, an example of the interaction of three factors (A, B and C) is shown, describing eight possible classes of casual mechanisms. The first class in this figure represents those mechanisms in which all the three components are involved. The U-named slice of the graph pie refers to an unknown causality component that interacts with A, B, and C in determining disease, as indicated by Rothman [7].

The partial configurations constitute the set of lower-order causal patterns that contribute to disease occurrence in the absence of complete exposure.

#### Role of $\:\boldsymbol{r}{\boldsymbol{D}}_{2,\boldsymbol{p}}^{*}$: dispositions with repetition

A central component of the generalized index is the term:

$$rD_{{2,p}}^{*}={2^p} - 2$$

which represents the number of dispositions with repetition of 2 elements taken * p* at a time, excluding the two extreme cases.

From a combinatorial perspective, dispositions with repetition count the number of ordered arrangements in which each of the * p* positions can take one of two values, with repetition allowed. In epidemiological terms:

- The two values correspond to exposure presence (1) or absence (0),
- Each position corresponds to one exposure factor.

By excluding the all-zero and all-one configurations, $\:r{D}_{2,p}^{*}$precisely enumerates the set of exposure patterns that represent incomplete causal constellations.

#### Epidemiological interpretation of $\:\boldsymbol{r}{\boldsymbol{D}}_{2,\boldsymbol{p}}^{*}$

Each of the $\:r{D}_{2,p}^{*}\:$partial exposure configurations corresponds to a distinct epidemiological scenario in which disease risk may be elevated due to:

- A single factor acting alone, or
- A subset of factors acting jointly, without full exposure.

These configurations collectively define the counterfactual expectation under an additive model of causation. Summing their excess risk therefore yields the expected joint effect if no higher-order interaction is present.

#### Definition of the generalized synergy index

The generalized Synergy Index for $\:p\:$factors is defined as:

$${S_p}=\frac{{R{R_{1 \ldots 1}} - 1}}{{\left( {\mathop \sum \nolimits_{{k=1}}^{{rD_{{2,p}}^{*}}} R{R_k}} \right) - rD_{{2,p}}^{*}}}$$

where:

- $\:R{R}_{1\dots\:1}$denotes the risk ratio associated with simultaneous exposure to all * p* factors;
- $\:R{R}_{k}$denotes the risk ratios associated with each of the $\:r{D}_{2,p}^{*}\:$partial exposure configurations.

This formulation constitutes a direct and natural extension of Rothman’s original index. When * p=2*, the generalized expression reduces exactly to Rothman’s two-factor formula, thereby ensuring theoretical continuity.

In other terms, our proposed generalization extends this logic to *n* exposures. The numerator represents the excess risk when all exposures are simultaneously present, and thus corresponds to the combined contribution of all sufficient causes that include the full set of exposures as component causes (i.e., causal pies requiring joint presence of all *n* factors). The denominator, in contrast, reflects the observed joint effect and includes all sufficient causes, namely:

- Pies involving a single exposure (*X*_*i*_ + *U*), and
- Pies involving any combination of exposures of size ≥ 2, i.e.,

$$(X_{1}+ \ldots +X_{k}+{\text{ }}U),{\text{ }}k\, \geqslant \,2.$$

#### Interpretation within the sufficient-cause framework

Within Rothman’s causal pie model, the generalized index can be interpreted as a comparison between:

- The excess risk generated by a complete causal pie, requiring all $\:p\:$components; and
- The cumulative excess risk generated by all incomplete pies, in which one or more components are missing.

A value of $\:{S}_{p}>1\:$therefore indicates that the full exposure constellation constitutes a qualitatively distinct sufficient cause, producing disease risk beyond what can be explained by any combination of partial exposures.

#### Estimation using logistic regression

To estimate the risk ratios required for the calculation of $\:{S}_{p}$, regression models are fitted including indicator variables for each joint exposure category, with the null configuration as the reference group.

This modeling strategy ensures that:

- Each risk ratio corresponds to a specific exposure constellation;
- The index is computed entirely on the additive scale of excess risk;
- Adjustment for confounders can be incorporated without altering the structure of the index.

### Data

We used a database of a previous work in which the synergy factor was calculated for each couples of two factors (alcohol, tobacco smoke and other risk factors) for age-related macular degeneration [8].

Effect estimates are presented as odds ratios (ORs). Although the synergy index is formally defined using risk ratios, ORs can be considered a reasonable approximation when the outcome is rare. This assumption is met in our study, and results should be interpreted accordingly.

We develop a logistic regression model with the following dichotomous variables:

Alcohol consumption (a), smoking (s) and familiarity for the AMD (f).

In the model we considered the OR of three factors combined as follows.

a1s1f1; a1s1f0; a1s0f0; a1s0f1; a0s0f1; a0s1f1; a0s1f0.

The model was adjusted for age and gender.

Following this approach the g-*S*_*p*_ was calculated using the following formula:

$$\begin{array}{cc} S_p = {\text{ }}({\text{OR a1s1f1}}{-}{\mathrm{1}})\;/[({\text{ OR a1s1f}}0\, + \,{\text{OR a1s}}0{\mathrm{f}}0\, \\ + \,{\text{OR a1s}}0{\mathrm{f1}}\, + \,{\text{OR a}}0{\mathrm{s}}0{\mathrm{f1}}\, + \,{\text{OR a}}0{\mathrm{s1f1}}\, + \,{\text{OR a}}0{\mathrm{s1f}}0){-}{\mathrm{6}}] \end{array}$$

In a second model, we introduced the hypercholestolemia (h) instead of the familiarity for AMD, so that the *S*_*p*_ was equal to (OR a1s1h1–1)/[( OR a1s1h0 + OR a1s0h0 + OR a1s0h1 + OR a0s0h1 + OR a0s1h1 + OR a0s1h0) – 6)].

The statistical analysis was carried out using SPSS (release 28.0).

## Results

### Joint effects of alcohol consumption, smoking, and family history of AMD (Table 1)

Table 1 presents adjusted odds ratios (ORs) for AMD across the eight possible joint exposure categories of alcohol consumption, smoking status, and family history of AMD, using the unexposed group (alc0_fam0_smoke0) as the reference category.

A clear gradient of increasing risk emerged across joint exposure categories, culminating in a markedly elevated odds of AMD among individuals simultaneously exposed to all three factors (alc1_fam1_smoke1). This triple-exposure category showed an adjusted OR = 77.636 (*p* < 0.001), indicating a very strong association between combined exposure and AMD occurrence. This finding emphasizes the relevance of capturing higher-order exposure patterns, as the joint effect substantially exceeds what would be inferred from examining single risk factors in isolation.

Several two-factor combinations were also associated with substantially increased odds. In particular, smoking combined with family history (alc0_fam1_smoke1) was associated with an OR of 11.516 (*p* = 0.004), suggesting an important amplification of smoking-related risk in individuals with familial susceptibility. This pattern supports the hypothesis that inherited or shared familial factors may increase biological vulnerability to environmentally induced retinal damage, thereby magnifying the effect of smoking. Smoking alone (alc0_fam0_smoke1) was associated with an OR of 3.521 (*p* = 0.001), confirming its role as a major modifiable risk factor for AMD. Alcohol combined with smoking in the absence of family history (alc1_fam0_smoke1) showed a comparable increase in risk (OR = 3.285, *p* = 0.001), suggesting that alcohol exposure may contribute to risk primarily through interaction with other behavioral factors rather than through a strong independent effect.

Family history in the absence of both alcohol and smoking exposure (alc0_fam1_smoke0) was associated with a pronounced elevation in odds (OR = 26.584, *p* = 0.007), underscoring the strong contribution of inherited or shared familial determinants to AMD risk. The magnitude of this association reinforces the conceptual role of family history as a proxy for complex genetic and early-life environmental influences that are not fully captured by individual behavioral variables. In contrast, alcohol consumption in isolation (alc1_fam0_smoke0) did not show a statistically significant association (OR = 1.358, *p* = 0.413), indicating that its epidemiologic relevance may be context-dependent and conditional on the presence of other risk factors.

Beyond the magnitude of individual joint-category estimates, the overall configuration of ORs in Table 1 reveals a coherent and biologically plausible interaction structure. Specifically, family history appears to function as a key susceptibility component that reshapes the joint-effect landscape, elevating risk across multiple exposure strata and markedly amplifying the impact of smoking when both are present. The pronounced increase in odds observed for the family-history-only category further suggests that familial susceptibility constitutes a strong underlying risk substrate upon which behavioral exposures can act. When smoking and alcohol are superimposed on this substrate, the resulting joint effect reflects not merely cumulative exposure but a qualitatively different risk profile. The exceptionally high OR associated with the triple-exposed category can therefore be interpreted as the epidemiologic manifestation of converging causal mechanisms, in which inherited vulnerability and modifiable behaviors jointly contribute to retinal degeneration through overlapping or mutually reinforcing pathways. Importantly, the progression of ORs across categories supports the internal consistency of the model: risk increases systematically as additional factors are introduced, with the steepest escalation occurring when family history and smoking co-occur, and the maximal elevation observed when all three factors are present. This ordered pattern strengthens confidence that the observed three-way synergism is not an artifact of isolated categories, but rather reflects a structured interaction architecture spanning the full exposure space.

Age was strongly and independently associated with AMD, with an OR of 1.110 per unit increase (*p* < 0.001), consistent with the well-established age dependence of the disease. Gender was not significantly associated with AMD in this model.

Using the proposed three-factor formulation, the Synergy Index for alcohol, smoking, and family history was calculated as follows.

$$\begin{array}{cc} S_p ={\text{ }}({\mathrm{77}}.{\mathrm{626}}-{\mathrm{1}})/{\text{ }}({\mathrm{3}}.{\mathrm{521}}\,+\,{\mathrm{11}}.{\mathrm{516}}\,+\,{\mathrm{3}}.{\mathrm{285}}\,+\,{\mathrm{1}}.{\mathrm{358}}\,+\, \\ {\mathrm{26}}.{\mathrm{584}}\, + \,{\mathrm{2}}.{\mathrm{7}}0{\mathrm{3}}-{\mathrm{6}}){\text{ }}=\,{\mathrm{1}}.{\mathrm{783}}\end{array}$$

Indicating the presence of a synergistic effect of the three factors combined. This value, exceeding unity, indicates positive three-way synergism on the additive scale, meaning that the combined effect of the three factors exceeds the sum of their individual and pairwise contributions.

Crucially, the *S*_*p*_ value integrates information from all joint exposure categories and formalizes the intuitive impression conveyed by the OR pattern in Table 1. By quantifying the extent to which the triple-exposure effect surpasses the composite expectation derived from lower-order combinations, the index confirms that family history, smoking, and alcohol consumption participate in a higher-order interaction that cannot be reduced to additive contributions alone. In this context, family history emerges as an “interaction-enabling” factor, transforming the joint impact of behavioral exposures and allowing them to combine in a super-additive manner. This insight highlights the added value of the proposed index in revealing aspects of disease causation that remain hidden when analyses are limited to main effects or pairwise interactions.

### Joint effects of alcohol consumption, smoking, and hypercholesterolemia (Table 2)

Table 2 reports adjusted ORs for AMD for the joint exposure categories of alcohol consumption, smoking, and hypercholesterolemia. The triple-exposed category (alc1_col1_smoke1) showed an adjusted OR = 6.319 (*p* < 0.001), indicating a statistically significant association between simultaneous exposure to these three factors and AMD.

Smoking emerged as the dominant contributor in this model. Smoking alone (alc0_col0_smoke1) was associated with an OR of 3.405 (*p* = 0.010), while smoking combined with hypercholesterolemia (alc0_col1_smoke1) yielded a similar OR of 3.209 (*p* = 0.020). The similarity of these estimates suggests that hypercholesterolemia does not substantially modify the smoking–AMD association in this dataset. Alcohol combined with smoking (alc1_col0_smoke1) was also associated with increased odds (OR = 2.362, *p* = 0.048). In contrast, alcohol consumption alone (alc1_col0_smoke0) and hypercholesterolemia alone (alc0_col1_smoke0) were not significantly associated with AMD.

Importantly, the joint-category profile observed in Table 2 provides a coherent epidemiologic picture in which smoking acts as the principal driver of elevated AMD odds, while hypercholesterolemia appears to contribute primarily as a background metabolic condition rather than as a strong independent determinant in this specific model. This pattern is informative because it suggests that the presence of hypercholesterolemia does not “distort” or obscure the smoking effect; instead, the smoking-related association remains stable across strata, supporting the interpretability and internal consistency of the fitted joint-effect model.

Moreover, the statistically significant elevation of the triple-exposed category (OR = 6.319) indicates that individuals accumulating multiple risk factors still experience a clearly higher odds of AMD compared with the fully unexposed reference group, even when the interaction structure is predominantly additive. In this sense, the model highlights that clinically relevant risk accumulation can occur without requiring super-additivity, and the proposed framework remains valuable because it distinguishes “risk accumulation” from “synergistic amplification.” From a mechanistic standpoint, these results are compatible with an interpretation in which smoking-related oxidative stress and vascular dysfunction constitute key pathways for AMD development, while hypercholesterolemia may intersect these pathways in a more heterogeneous or indirect manner, contributing to overall cardiovascular-metabolic burden but not necessarily producing an additional amplification of the smoking effect in the current exposure definition. In addition, the distribution of ORs across categories suggests that the combination of alcohol and smoking (OR = 2.362) yields a detectable increase in odds relative to the reference, reinforcing the concept that behavioral co-exposures can form meaningful joint profiles. The fact that the triple combination is higher than this two-factor combination further supports the epidemiologic relevance of considering multi-factor constellations in AMD, even when the departure-from-additivity metric does not indicate synergism.

The pattern of estimates also strengthens the interpretive role of the Synergy Index: rather than simply mirroring the magnitude of OR_{111}, SI contextualizes the triple-exposure estimate against the “expected” composite contribution of the other joint categories. Consequently, the *S*_*p*_ < 1 observed here can be viewed as an informative descriptor of the interaction architecture—indicating that the observed triple-exposure elevation is largely accounted for by the contribution of smoking-driven and lower-order joint effects—while still acknowledging that the triple-exposed group constitutes a higher-risk phenotype. Finally, these findings contribute to the comparative objective of the study: by holding alcohol and smoking constant and substituting family history with hypercholesterolemia, Table 2 illustrates how different third factors can yield distinct multi-way joint-effect configurations. This emphasizes the practical utility of the proposed index not only for detecting synergy, but also for characterizing and contrasting higher-order risk structures across alternative etiologic hypotheses.

Age again showed a strong independent association with AMD (OR = 1.106, *p* < 0.001), while gender was not statistically significant. The Synergy Index for alcohol, smoking, and hypercholesterolemia calculated as follows:

$$\begin{array}{cc} S_p ={\text{ }}({\mathrm{6}}.{\mathrm{319}}-{\mathrm{1}})/{\text{ }}({\mathrm{1}}.0{\mathrm{68}}\,+\,{\mathrm{1}}.{\mathrm{665}}\,+\,{\mathrm{2}}.{\mathrm{362}}\,+ \\ \,{\mathrm{3}}.{\mathrm{4}}0{\mathrm{5}}\,+\,{\mathrm{3}}.{\mathrm{2}}0{\mathrm{9}}\,+\,{\mathrm{1}}.{\mathrm{326}}-{\mathrm{6}})=\,0.{\mathrm{756}} \end{array}$$

indicating no evidence of positive three-way synergism on the additive scale. In this example alcohol, tobacco and hypercholesterolemia tend to act independently, i.e. through separate causal mechanisms, as opposed to a single mechanism involving all three factors. This finding suggests that, unlike family history, hypercholesterolemia does not act as a major susceptibility amplifier within this specific three-factor configuration.

**Table 1:** Results of the logistic regression analysis using the variables alcohol, smoking and familiarity for AMD

	B	S.E.	Wald	df	Sign.	Exp(B)		
alc1_fam1_smoke1	4,352	1,148	14,381	1	000	77,636	8,189	736,042
alc0_fam0_smoke1	1,259	374	11,301	1	001	3,521	1,690	7,335
alc0_fam1_smoke1	2,444	847	8,317	1	004	11,516	2,188	60,611
alc1_fam0_smoke1	1,189	355	11,223	1	001	3,285	1,638	6,587
alc1_fam0_smoke0	306	374	671	1	413	1,358	653	2,826
alc0_fam1_smoke0	3,280	1,206	7,394	1	007	26,584	2,499	282,820
alc1_fam1_smoke0	994	842	1,394	1	238	2,703	519	14,087
Age	105	014	57,190	1	000	1,110	1,081	1,141
Gender	285	251	1,292	1	256	1,329	814	2,172
Constant	-8,982	1,062	71,464	1	000	000		

**Table 2:** Results of the logistic regression analysis using the variables alcohol, smoking and hypercolesterolomia

	B		S.E.		Wald		df		Sign.		Exp(B)			
alc1_col1_smoke1	1,844		484		14,497		1		000		6,319		2,446	16,322
alc1_col0_smoke0	066		471		019		1		889		1,068		424	2,690
alc1_col1_smoke0	510		525		942		1		332		1,665		595	4,662
alc1_col0_smoke1	859		434		3,927		1		048		2,362		1,009	5,525
alc0_col0_smoke1	1,225		477		6,607		1		010		3,405		1,338	8,669
alc0_col1_smoke1	1,166		501		5,411		1		020		3,209		1,201	8,569
alc0_col1_smoke0	282		479		347		1		556		1,326		519	3,388
Age	100		014		54,530		1		000		1,106		1,076	1,135
Gender	251		245		1,048		1		306		1,285		795	2,076
Constant	-8,504		1,049		65,775		1		000		000			

### Comparative interpretation of three-factor configurations

Taken together, the results demonstrate that the proposed three-factor SI is sensitive to the underlying structure of the risk factors considered. When a strong susceptibility component such as family history is included, the combined exposure exhibits clear super-additive behavior. Conversely, when hypercholesterolemia replaces family history, the joint effect remains substantial but does not exceed additivity.

This contrast illustrates how the index can discriminate between exposure sets that participate in higher-order interaction mechanisms and those that operate predominantly through lower-order effects.

Beyond this empirical contrast, the comparative analysis of the two three-factor configurations provides important conceptual insights into the nature of multifactorial disease causation. In particular, the results suggest that not all risk factors contribute symmetrically to higher-order interactions: certain factors, such as family history, may function as “interaction-enabling” components that unlock or potentiate the effects of co-occurring exposures. In this sense, the presence of a familial susceptibility background appears to modify the overall causal architecture, allowing behavioral exposures to combine in a manner that exceeds additivity.

The markedly different SI values observed across the two models indicate that the proposed index is capable of capturing qualitative differences in interaction structure, rather than merely reflecting differences in the magnitude of individual associations. This feature is especially relevant in complex diseases like AMD, where etiologic pathways are heterogeneous and where certain exposures may act primarily as effect modifiers rather than as strong independent causes.

Furthermore, the comparative findings highlight the importance of explicitly evaluating alternative multi-factor configurations within the same dataset. By holding two exposures constant (alcohol and smoking) and varying the third, the analysis demonstrates how the nature of the accompanying factor can fundamentally alter the joint effect profile. This approach moves beyond traditional pairwise interaction analyses and enables a more nuanced exploration of how different combinations of risk factors jointly shape disease risk.

From a broader epidemiologic perspective, these results reinforce the value of higher-order interaction metrics in distinguishing between additive, synergistic, and context-dependent risk patterns. The ability of the three-factor SI to identify super-additivity in one configuration but not in another underscores its potential utility as a comparative tool for prioritizing biologically and clinically meaningful exposure constellations.

Overall, the comparative interpretation of the two three-factor models illustrates that the generalized *S*_*p*_ is not merely a numerical extension of existing indices, but a framework that supports deeper inference about the structure of multifactorial risk. By revealing which combinations of exposures are more likely to participate in synergistic mechanisms, the index contributes to a more refined understanding of disease causation and offers a principled basis for hypothesis generation in future etiologic research.

It is noteworthy that, in the original dataset, the synergy index (SI) exceeded 1 for the following pairs of exposures, namely family history of AMD and smoking (SI = 1.51), hypercholesterolemia and smoking (SI = 1.77), and hypercholesterolemia and alcohol consumption (SI = 1.64). In contrast, when considering three-way interactions, an SI greater than 1 was observed only for the combination of alcohol consumption, smoking, and family history of AMD.

## Discussion

The objective of this study was to perform a generalization of the Rothman’s approach for three or more factors considered simultaneously. In particular our attempt was to use in the analysis three factors simultaneously.

In this study, we introduced and applied a generalized Synergy Index for three simultaneous dichotomous factors, extending classic additivity-based interaction concepts to higher-order exposure constellations. The empirical application to AMD demonstrates that this approach yields meaningful and interpretable insights into complex disease etiology.

The analysis revealed a strong three-way synergistic effect among alcohol consumption, smoking, and family history of AMD, as indicated by an SI substantially greater than one.

This result supports a conceptual model in which genetic or familial susceptibility enhances the pathogenic impact of behavioral exposures, leading to a combined effect that is greater than the sum of its parts. Such a pattern is consistent with current biological understanding of AMD as a disease driven by the interplay between inherited vulnerability and cumulative environmental stressors.

In contrast, the absence of synergism in the model including hypercholesterolemia indicates that not all combinations of established or suspected risk factors necessarily interact in a super-additive manner.

This distinction underscores the importance of formally evaluating higher-order interactions rather than assuming their presence based solely on individual associations. The proposed index provides a structured framework for making such evaluations explicit and comparable across different exposure sets.

This approach is original among epidemiological studies. There is no single universally accepted method to evaluate synergy among more than two factors, even if there are complementary approaches that, when well-designed, can yield meaningful insights. In fact, usually statistical models with Multi-way Interaction Terms in regression models could include three-way interaction terms (e.g., A*B*C) to assess whether the combined effect of three factors exceeds the sum of their individual effects.in this case, the interpretation becomes complex and unstable, in particular with small sample sizes.

For the assessment of the effect of multiple factors on a specific outcome, there are also several computational epidemiology approaches, that include techniques such as Bayesian models, machine learning, and agent-based simulations. These methods are really promising but require detailed data and rigorous validation. Moreover, using these methods we cannot solve the problem of the synergistic effect.

Moreover, Hoffman and coll. [9] tried to assess the synergistic effect on an additive scale, using the attributable risk, or multiplicative scale, using odds ratios. These approaches are used to extend Rothman’s model by attempting to *quantify* the proportion of disease attributable to combinations of risk factors.

For more than two factors, hierarchical models or causal networks (e.g., DAGs—Directed Acyclic Graphs) can help explore interactions [10–11].

From a methodological perspective, these findings highlight the practical utility of the three-factor SI as a summary measure of interaction that can be derived directly from standard regression outputs.

By condensing complex joint-exposure information into a single interpretable parameter, the index facilitates both etiologic interpretation and communication of results, particularly in studies of multifactorial diseases.

Looking ahead, several avenues for further development emerge naturally from this work.

Future research may extend the index to accommodate non-binary exposures, explore its behavior in longitudinal designs, or integrate it within causal modeling frameworks. Application of the approach to larger datasets and to other complex diseases may further clarify its generalizability and potential role in epidemiologic inference.

## Conclusions

In conclusion, the generalized multi-factor Synergy Index (*S*_*p*_) represents a valuable conceptual and analytical tool for investigating higher-order interactions.

Its application to AMD illustrates how explicitly modeling and quantifying multi-factor synergy can deepen understanding of disease causation beyond traditional single-factor analyses.

## References

[1] CCOHS. Synergism and related terms. 2019. Available at https://www.ccohs.ca/oshanswers/chemicals/synergism.pdf, Accessed 15 Jan 2026.

[2] Rothman KJ. Causes. Am J Epidemiol. 1976;104:587–92. 998606 10.1093/oxfordjournals.aje.a112335

[3] Rothman KJ. Synergy and antagonism in cause-effect relationships. Am J Epidemiol. 1974;99(6):385–8. 4841816 10.1093/oxfordjournals.aje.a121626

[4] Rothman KJ. The estimation of synergy or antagonism. Am J Epidemiol. 1976;103(5):506–11. 1274952 10.1093/oxfordjournals.aje.a112252

[5] Rothman KJ, Greenland S, Walker AM. Concepts of Interaction. Am J Epidemiol. 1980;112(4):467–70. 7424895 10.1093/oxfordjournals.aje.a113015

[6] Cortina-Borja M, Smith AD, Combarros O, et al. The synergy factor: a statistic to measure interactions in complex diseases. BMC Res Notes. 2009;2:105. 19527493 10.1186/1756-0500-2-105 PMC2706251

[7] Rothman KJ. Epidemiology: An Introduction. Oxford University Press; 2002.

[8] La Torre G, Pacella E, Saulle R, Giraldi G, Pacella F, Lenzi T, Mastrangelo O, Mirra F, Aloe G, Turchetti P, Brillante C, De Paolis G, Boccia A, Giustolisi R. The synergistic effect of exposure to alcohol, tobacco smoke and other risk factors for age-related macular degeneration. Eur J Epidemiol. 2013;28(5):445–6. 23543124 10.1007/s10654-013-9798-7

[9] Hoffmann K, Heidemann C, Weikert C, Schulze MB, Boeing H. Estimating the proportion of disease due to classes of sufficient causes. Am J Epidemiol. 2006;163(1):76–83. 10.1093/aje/kwj011. Epub 2005 Nov 17. PMID: 16293718. 16293718

[10] Rodrigues D, Kreif N, Lawrence-Jones A, Barahona M, Mayer E. Reflection on modern methods: Constructing directed acyclic graphs (DAGs) with domain experts for health services research. Int J Epidemiol. 2022;51(4):1339–48. 35713577 10.1093/ije/dyac135 PMC9365627

[11] Tennant PWG, Murray EJ, Arnold KF, Berrie L, Fox MP, Gadd SC, Harrison WJ, Keeble C, Ranker LR, Textor J, Tomova GD, Gilthorpe MS, Ellison GTH. Use of directed acyclic graphs (DAGs) to identify confounders in applied health research: review and recommendations. Int J Epidemiol. 2021;50(2):620–32. 33330936 10.1093/ije/dyaa213 PMC8128477

